# A SYSTEMATIC REVIEW OF MEASURES OF SOCIAL CONNECTION FOR PEOPLE LIVING IN LONG-TERM CARE HOMES: THE SONNET STUDY

**DOI:** 10.1093/geroni/igad104.1849

**Published:** 2023-12-21

**Authors:** Jennifer Bethell, Andrew Sommerlad, Hannah Chapman, Neha Dewan, Madalena Liougas, Gill Livingston, Katherine McGilton

**Affiliations:** Toronto Rehabilitation Institute - University Health Network, Toronto, Ontario, Canada; University College London, London, England, United Kingdom; University College London, London, England, United Kingdom; Toronto Rehabilitation Institute - University Health Network, Toronto, Ontario, Canada; University of Toronto, Toronto, Ontario, Canada; University College London, London, England, United Kingdom; Toronto Rehabilitation Institute - University Health Network, Toronto, Ontario, Canada

## Abstract

Social connection is important for quality of life and care in long-term care (LTC) homes. However, measuring social connection in LTC residents is challenging and research is limited by a lack of consensus on best approaches to measurement. Our study objective is to systematically review measures of social connection developed for LTC residents. We are following COnsensus-based Standards for the selection of health Measurement INstruments (COSMIN) systematic review methods. We searched multiple bibliographic databases to April 2022 for studies conducted in LTC residents that quantified any aspect(s) of social connection and reported at least one psychometric property for the measure(s) of social connection. We are using COSMIN guidelines to evaluate the properties reported for each measure. We identified 60 studies reporting on 38 measures that assess social connection in LTC homes. Twenty-eight were measures of quality of life, wellbeing or life satisfaction and included a social connection subdomain. Only 10 measures specifically targeted social connection. From our evaluation of 32 measures to date, we found that 16% (n=5) have sufficient structural validity, 9% (n=3) have sufficient internal consistency, 22% (n=7) have sufficient reliability, and 19% (n=6) have sufficient construct validity. Many measures have assessed social connection in LTC homes, but few are designed for this purpose and many have insufficient psychometric properties. This review will provide detailed evidence of the quality of these measures to enable researchers to prioritise valid and reliable tools. Our results will inform our development of a new person-centred measure of social connection for LTC residents.

